# Rectal Signet Ring Cell Carcinoma: Post-Chemoradiotherapy Evaluation by MRI and Corresponding to Pathology

**DOI:** 10.3389/fsurg.2022.841645

**Published:** 2022-03-04

**Authors:** Yin Zhou, Qingshu Li, Yun Mao

**Affiliations:** ^1^Department of Radiology, The First Affiliated Hospital of Chongqing Medical University, Chongqing, China; ^2^Department of Pathology, School of Basic Medicine, Chongqing Medical University, Chongqing, China

**Keywords:** signet ring cell carcinoma, colorectal carcinoma, magnetic resonance imaging, chemoradiotherapy, pathology

## Abstract

**Background:**

Signet ring cell carcinoma (SRCC) is recognized as an uncommon subtype of colorectal carcinoma (CRC). It showed characteristic magnetic resonance imaging (MRI) manifestations. However, the MRI features post-chemoradiotherapy (CRT) were not reported, and it is unknown whether the current tumor regression grade (TRG) system by MRI (mrTRG) is applicable to SRCC.

**Purpose:**

To summarize the image features of rectal SRCC on post-CRT images corresponding to the pathology, and to determine the predicting value of mrTRG compared with TRG by pathology (pTRG).

**Methods:**

We retrospectively enrolled seven patients (male: female = 3:4; mean age, 45.1 years) with biopsy-pathology proved SRCC, who underwent pre- and post-CRT MR imaging followed by surgery. An experienced gastrointestinal radiologist accessed mrTRG using a 5-point grading system by mandard standard on T2 weighted image (T2WI) and then added diffusion weighted image (DWI) in a 1-month interval. Additionally, MRI features were recorded on pre- and post-CRT images as follows: pattern (target sign) and main signal intensity of T2WI, characterized manifestation of DWI, and mean Apparent Diffusion Coefficient (ADC)values. The mrTRG and all MR image features were compared to the post-operative pathology.

**Results:**

At post-CRT histology, five patients got a good response (TRG 1, n = 4; TRG 2, n = 1), one patient got a partial response, and one patient got a poor response. The accuracy of MRI predicted the pathology response by mandard standard was 14% and increased to 71.4% when added DWI. After CRT, different degrees of homogeneous high SI without enhancement representing acellular mucin were observed in all patients, and the thick-ring high SI turned into a thin-target sign in most good responders. Moreover, the tumor volume decreased or slightly increased in good responders, while it markedly increased in the partial and poor responder by 57% and 73.8%, respectively.

**Conclusion:**

Homogeneous high SI on T2WI and thin target sigh on DWI were the main MRI changes of RSRCC, which was corresponding to the mucinous regression and represents for good response post-CRT. The mrTRG and tumor volume was not a reliable indicator to the pathology response. We considered that DWI should be added to T2WI to evaluate RSRCC response to CRT.

## Introduction

Signet ring cell carcinoma (SRCC) is defined as a carcinoma composed of >50% of signet ring cells (SRCs) by the World Health Organization (WHO) (1). In comparison, cases with ≤50% presence of SRCs are noted to be carcinomas with SRCs. SRCC shows more aggressive behavior and a worse clinical outcome than adenocarcinoma (AC) and mucinous adenocarcinoma (MAC) (2–4), even than MAC with SRCs (5, 6). Rectal SRCC (RSRCC) patients presented more frequently with Stage III or IV disease than other subtypes. Pre-operative chemoradiotherapy (CRT) followed by total mesorectal excision (TME) has become a standard treatment for patients with locally advanced rectal cancer. The patients with stage III RSRCC treated with pre-operative radiotherapy followed by surgery had better cause-special survival than that with surgery alone (7). In addition, Bratland et al. (8) reported that half of six patients with RSRCC turned into complete pathological response (pCR), and half had no response after radiotherapy. So the precise evaluation of the tumor regression post-CRT is required to determine further therapeutic strategies.

MRI is routinely applied for assessing the pre-operative stage and treatment response to pre-operative CRT. However, the previous studies almost focused on the AC or MAC (9–11). AC manifested as an isointense solid mass with diffusion restriction. The tumor regression was recognized as tumor mainly replacing by fibrosis, which showed hypointensity on T2WI, with markedly volume shrinkage and without diffusion restriction (12, 13). MAC manifested as a high signal intensity mass with multiple isointense tumor foci, corresponding to large extracellular mucin lined by columns of malignant cells, cords, and vessels. The tumor regression of MAC depended on the tumor foci pattern, which showed similar changes with AC. Disappearance of the tumor foci manifests homogeneous high SI without enhancement, which indicated good response. And only minimal volume shrinkage was observed due to the constancy existing of the extracellular mucin. However, few case reports depicted SRCC as concentric wall thickening with target sign and low SI on T2WI due to extensive scatter malignant cells, with intracellular mucin separating the anatomical layers of the rectal wall (14). It shows a big difference in histopathology and imaging features from AC and MAC. Its MR manifestations of post-CRT had not been reported, so it is unknown whether the current mrTRG system is applicable to SRCC.

In this retrospective study, we attempted to reveal the MR characteristics changes of RSRCC post-CRT, corresponding to post-operative pathology, and obtain a preliminary evaluation of association MR features to pathology tumor regression.

## Materials and Methods

### Patients and Clinical Information

This retrospective study was approved by ethics committee of the first affiliated hospital of Chongqing Medical University. Thirty-one consecutive patients with pathology proved rectal carcinoma with an SRC component were identified from the electronic clinical database in our hospital between January 2012 and March 2021. The patients were included by the following criteria: (a) patients who had locally advanced rectal cancer and underwent neoadjuvant CRT followed by surgery within 6 weeks; (b) patients who underwent pelvic MR scanning pre- and post-CRT. Exclusion criteria: (a) patients with secondary rectal SRCC (*n* = 1, from gastric SRCC); (b) patients with primary rectal cancer did not undergo surgery in our hospital (*n* = 9) or neoadjuvant CRT (*n* = 9); (c) the pre or post-CRT MRI was unavailable (*n* = 3); (d) the biopsy pathology patients did not meet the SRCC criteria by WHO (*n* = 2). Finally, seven patients were enrolled.

The clinical data of the enrolled patients were also recorded, including age, sex, colonoscopy and biopsy result, and CRT strategies.

### MR Imaging Method

MRI was performed with a 3.0-T MR imager (Signa HDxT, GE) and a pelvic phased-array coil. The same protocol was used in every time MR examination in all patients, which included the following sequences at least: an oblique axial, coronal, and sagittal T2 weighted fast spin-echo sequence without fat suppression (TR, 2,320–3,120 ms; TE, 85–110 ms; flip angle, 90°; matrix, 512 × 512; NEX, 2; slice thickness, 3 mm); an oblique axial diffusion-weighted sequence with fat suppression (TR, 4,925–6,200 ms; TE, 64–71 ms; flip angle, 90°; matrix, 256 × 256; NEX, 4–5; slice thickness, 4 mm; *b* = 0, 600); an axial, coronal, and sagittal LAVA sequence after contrast was injected (TR, 4.27–4.66 ms; TE, 2.08–2.22 ms; flip angle, 15°; matrix, 512 × 512; slice thickness, 4 mm). The Gadopentetate Dimeglumine (Magnevist; Bayer Schering Pharma AG, Germany) was injected into the elbow vein as a contrast agent.

### Imaging Analysis

**Figure 1:**
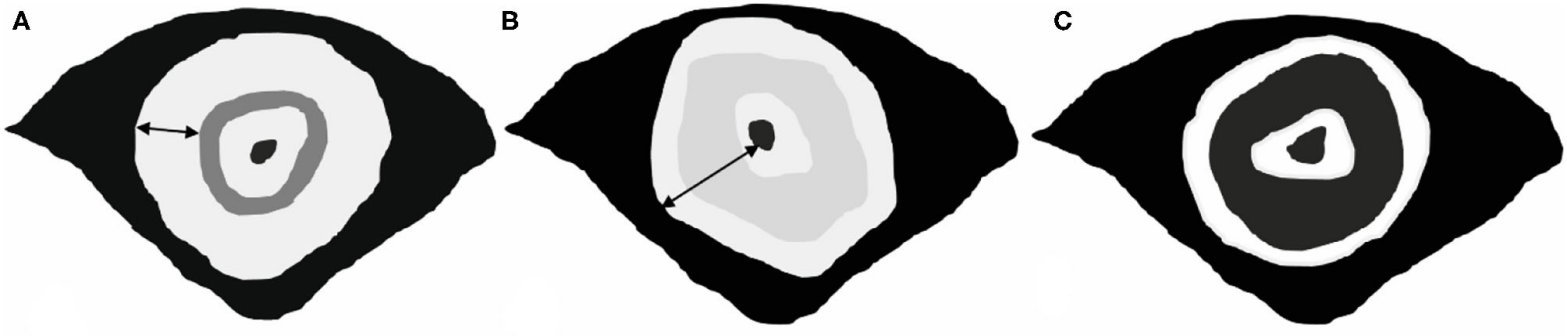
Patterns of SRCC on DWI. **(A,B)** Definition of thick-ring signs. The wide of the high-SI ring is above 5 mm, which represents tumor tissue infiltrated all mural layers. **(C)** Definition of a thin-target sign. The thick concentric mural with high SI of thin inner and outer layers (<5 mm) and low SI of the middle layer, which was assumed non-tumor mural.

A gastrointestinal radiologist with 12 years of experience in abdominal MR imaging (Mao Yun, an associate professor), who was aware of biopsy results and treatment protocol but blind to the post-operative histopathology and other clinical information analyzed all images independently, firstly for T2WI of pre and post-CRT, 1 month later for T2WI with DWI. MR image features were recorded on pre and post-CRT images as follows: pattern (target sign or non-target sign) and main signal intensity (low, intermediate high, or high) of T2WI, characterized manifestation of DWI, mean ADC value, MR T stage, MRF involvement, and EMVI. Patterns of T2WI were evaluated on oblique axial images. Low SI was defined as lower or similar to the skeletal muscle, intermediate high SI as slightly higher than skeletal muscle, and high SI as markedly higher than skeletal muscle. On DWI, the thin-target sign was defined as a thick concentric mural characterized by high SI of thin inner and outer layers (<5 mm) and low SI of the middle layer, which was assumed non-tumor mural. A thick ring sign was noted in if the middle layer showed high SI or if the high SI layer thickened above 5 mm, which was hypothesized mural with tumor infiltrated (Figure 1). The ROI on the ADC map was drawn along the contour of the tumor area on each slice on the workstation, and the mean ADC value was finally recorded. The change of ADC value (δ ADC) was calculated as _post_ADC value-_post_ADC value. The T stage accessed at MR imaging based on the depth of tumor or mucin involving: T2, confined to muscularis propria; T3, extended to peripheral adipose tissue; T4, involved the peritoneal reflection (T4a)/extended to adjacent organs (T4b). Negative MRF was defined as the interval between the outermost tumor tissue and MRF above 1 mm. Otherwise, it recorded positive MRF. The EMVI was defined as either intermediate or high SI was observed within vessels outside the tumor, or the contours of these vessels appeared irregular. ymrTRG was evaluated post-CRT using the 5-point-grade system on T2WI (mandard standard): Grade 1, no evidence of treated tumor; Grade 2, dense hypointense fibrosis or acellular mucin with minimal residual tumor; Grade 3, >50% fibrosis or mucin with an intermediate tumor signal representing residual tumor (fibrosis and mucin > tumor); Grade 4, minimal fibrosis or mucinous degeneration, but mostly tumor (15); and Grade 5, the tumor that has a similar appearance as the baseline. We assumed the low, intermediate high, and high T2-weighted SI representing fibrosis, tumor, and mucin, respectively, while high SI without enhancement indicated the acellular mucin. After 1 month, DWI patterns were added to access the ymrTRG compared to pre-CRT images; the area of diffused high SI through the mucosa to the out layer was assumed as residue tumor, and the stratified pattern with low SI in the middle layer was defined as tumor regression completely.

Tumor volumes were computationally calculated by adding the ROI of consecutive images, and the ROI of the tumor area was drawn along the border of the tumor on each slice of oblique axial T2WI. The reductive ratio was calculated as [(volume_pre_-volume_post_)/volume_pre_] × 100%.

### Histopathology and Evaluation of Tumor Regression

HE stain was applied for all the pathology specimens, and the samples contained all tumor and regression tissue we suspected. A pathologist with 10 years of experience (Qingshu Li, an associate professor) in gastrointestinal pathology who was aware of the biopsy results but blind to the ymrMR result evaluated the tumor regression grade by mandard standard (16). The distribution of tumor or regression tissue in the mural, resection margin, LN involvement, extramural venous invasion, and invasion to other organs was also evaluated. TRG 1 and 2 scores were considered good responses, the TRG 3 score was a partial response, and TRG 4 and 5 scores were poor responses.

## Results

### Patients and Clinical and Histological Information

All enrolled seven patients included three men and four women (median age, 45.1 years; age range, 37–63 years). The percentage of SRC among the tumor of biopsy specimens was all above 50% (87%, 55–100%). They all accepted pelvic radiotherapy and synchronal chemotherapy due to locally advanced rectal cancer and positive CRM evaluated by MRI. A total dose of 50–56 Gy was administered to the pelvis in 25–28 daily fractions during 5 weeks (2 Gy/day, 5 days/week). Chemotherapy was applied with XELOX (*n* = 6) or FOFIRI (*n* = 1) regimen for 2–5 cycles. The mean interval between completed CRT and post-MRI was 4–6 weeks (34.5 ± 5.4 days). The interval between post-MRI and surgery was 11.7 ± 13.2 days. Surgical procedures included Dixon (*n* = 3), Miles (*n* = 3), and TME with partial bladder resection and lateral lymph node resection (*n* = 1), based on the post-CRT MRI evaluation of a tumor site and extension.

At post-CRT histology, most tumors of the rectal mural met complete pathological response (*n* = 4, TRG 1), and one patient showed few residue tumors (TRG 2). They were considered good responders. The poor and partial responders with positive MRF were noted in one case, respectively. Mucinous regression occurred in all tumors, and numerous inflammatory cells, especially lymphocytes and some collagen fiber, coexisted in the submucosa or outer membrane of only two patients. Fibrous tissue containing extensive scatter SRCs with little mucin was observed in the poor responder with low SI of T2WI. Three (43%) of seven patients did not show inflammatory and desmoplastic reactions. Five (71.4%) of seven cases showed a concentric thickening mural with its layers widened by the infiltrated tumor cells or regressed tissue without losing the rough contour. The submucosa and muscularis proporia were mainly involved. The circular muscle (inner muscularis) predominately widened, followed by longitudinal muscle (outer muscularis), which resulted in the muscle bundles detached and fence-like form, while a lot of mass-like mucin pool in pathology was observed in the two patients showed MAC-like form on MRI.

### Tumor Characteristics Pre- and Post-CRT on MRI

All cases manifested circular thickened rectal mural, and the mean volume before CRT was 56.8 ± 30.6 cm^3^. After CRT, tumor volume decreased or slightly increased in good responders, while it markedly increased in the partial responder and the poor responder by 57 and 73.8%, respectively. The tumor shrank most in the patients with a much poorly differentiated adenoma component.

**Figure 2:**
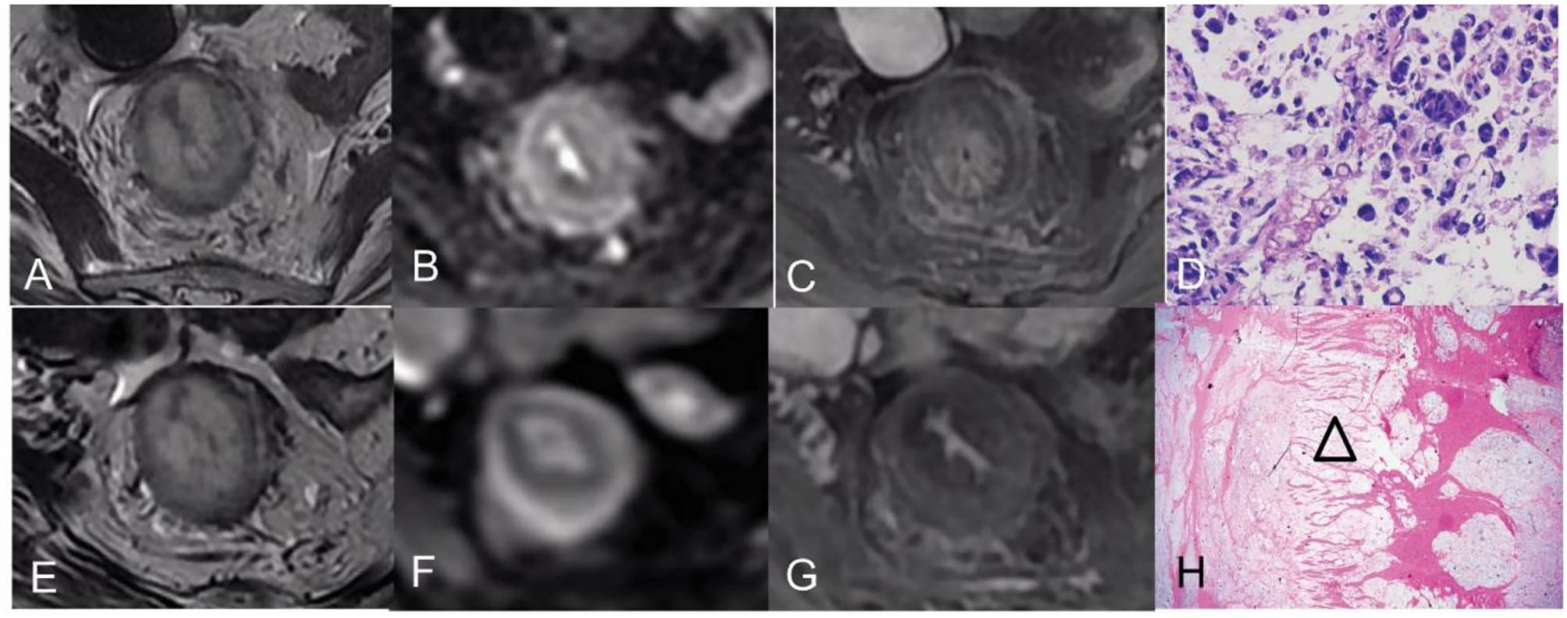
Pre- and post-CRT rectal MR images and pathology images of a 46-year-old woman [good responder (1) with RSRCC]. **(A–C)** axial T2WI, DWI, and contrast-enhanced T1WI pre CRT, the rectal mural showed a target sign with high SI on T2WI **(A)**, a thick ring sign with slightly high SI on DWI **(B)**, and persistent enhancement of the entire mural layers **(C)**. **(D)** The biopsy showed 100% of SRCs (Hematoxylin and eosin staining, × 400). **(E–G)** Axial T2WI, DWI, and delayed contrast-enhanced T1WI post-CRT, the lesion showed similarly to that of pre CRT on T2WI **(E)**, which resulted in mrTRG 5 in the evaluation of CRT response. However, the thin target sign on DWI indicated complete response **(F)**, and the decreased enhancement of the mural also indicated a good response **(G)**. **(H)** An extensive mucin pool infiltrated the mural layers with detached muscular bands (an empty triangle) (Hematoxylin and eosin staining, × 20).

**Figure 3:**
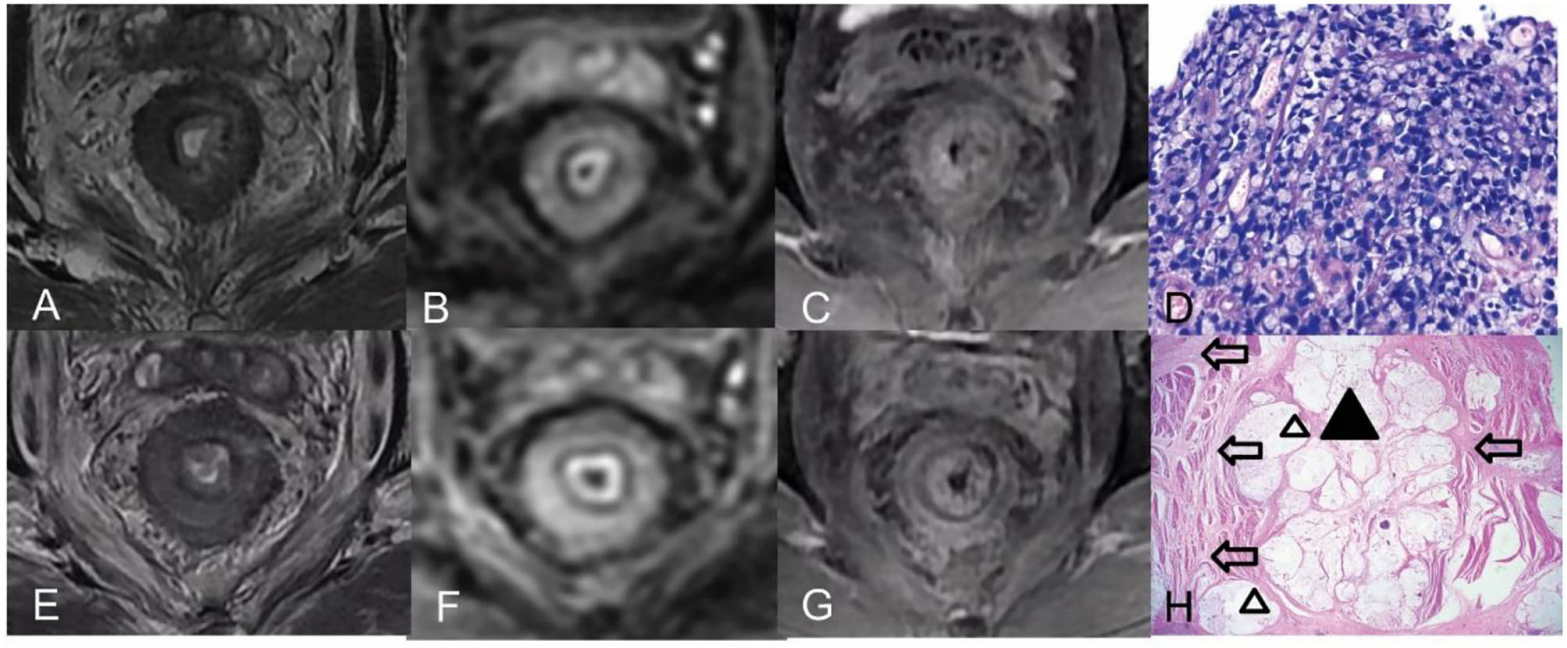
Pre- and post-CRT rectal MR images and pathology images of a 42-year-old man (a poor responder) with RSRCC. **(A–C)** Axial T2WI, DWI, and contrast-enhanced T1WI pre CRT, the rectal mural showed a target sign with low SI on T2WI **(A)**, a thick ring sign with high SI on DWI **(B)**, and persistent enhancement of the entire mural layers **(C)**. **(D)** of the biopsy showed 100% of SRCs (Hematoxylin and eosin staining, × 400). **(E–G)** Axial T2WI, DWI, and delayed contrast-enhanced T1WI post-CRT, the lesion showed a target sign with moderate SI on T2WI with a little mucin pool on T2WI **(E)**, which resulted in mrTRG 4 in the evaluation of CRT response. **(F,G)** DWI and an enhancement pattern showed similar to the pre CRT, which indicated poor response. **(H)** Extensive SRCs (a black triangle) infiltrated the mural layers with detached muscular bands with fibrosis (empty arrows) and mucinous degeneration (empty triangles) (Hematoxylin and eosin staining, × 20).

The majority of cases presented the target sign with multiple discontinuous rings on T2WI (five of seven), but different SI was observed among the cases (high SI, *n* = 2; moderate SI, *n* = 2; low SI, *n* = 1) pre CRT; they showed diffused or partial thick-ring-high SI on DWI. However, the other two patients mimicked mucinous adenocarcinoma without a target sign on T2WI and scattered-high SI on DWI. The mean pre-ADC value of all tumors was 1.37 ± 0.18 × 10^−3^ mm^2^/s. After CRT, the targetoid patterns of tumor on T2WI did not change, but different levels of the homogeneous high-SI area without enhancement, which represented a mucin pool, (Figure 2) was observed in all the patients. The low T2-SI of the poor responder turned into intermediate-high tumor SI. On post-CRT DWI, almost all good responders (4/5) turned into a thin target sign, and the ADC value increased, while one good responder and the partial/poor responder showed almost the same, comparing to pre CRT (Figure 3), and the ADC value changed slightly. The δADC value of good responders (0.346 ± 0.201 × 10^−3^ mm^2^/s) was higher than partial/poor responders (−0.100 ± 0.028 × 10^−3^ mm^2^/s). The pre- and post-CRT MRI features and pathology changes of all cases are shown in Table 1.

**Table 1:** The main clinical, image features, and pathology information pre and post-CRT.

		**Good-responder1**	**Good-responder2**	**Good-responder3**	**Good-responder4**	**Good-responder5**	**Partial-responder**	**Poor-responder**
Age/sex		46/F	46/M	41/F	41/M	63/F	37/F	42/M
Pre-CRT pathology and MRI	Biopsy pathology (SRCs %)	100	100	60, with poorly differentiated AC	95, with poorly differentiated AC	55, with MAC	100	100
	T2-pattern/SI	Target sign/high	Target sign/high	Target sign/intermediate high	Target sign/intermediate high	Non-target sign/high	Non-target sign/high	Target sign/low
	Enhanced pattern	Diffused enhancement	Diffused enhancement	Diffused enhancement	Diffused enhancement	Diffused enhancement	Diffused enhancement	Diffused enhancement
	DWI pattern	Diffuse/Thick ring sign/slightly high	Diffuse /Thick ring sign/slightly high	Partial/thick ring sign/high	Partial/thick ring sign/slightly high	Scatter high	Scatter high	Thick ring sign/ high
Post-CRT MRI	T2-SI	No change	Extensive acellular mucin pool	Partial fibrosis and acellular mucin pool	Partial acellular mucin pool	Partial acellular mucin pool	Partial acellular mucin pool and fibrosis	Focal acellular mucin pool
	Enhanced pattern	Markedly decrease	Markedly decrease	Partial decrease	Partial decrease	Markedly decrease	Partial decrease	No change
	DWI pattern	Thin targetoid	Thin targetoid	thin targetoid with little scatter high	Partial/thick ring sign/slightly high	Thin targetoid	Thin targetoid with little scatter high	Thick ring sign/ high
	δVolume (%)	−27%	+6.4%	−66%	+18.3%	−35.6%	+57%	+73.8%
	δADC (× 10^−3^ mm^2^/s)	+0.46	+0.39	+0.43	−0.01	+0.46	−0.03	+0.01
Post-CRT pathology	Main detached layers	Propria muscularis	Submucosa and propria muscularis	Submucosa and propria muscularis	Circular muscle	Circular muscle	Submucosa and propria muscularis	propria muscularis
	Regression tissue	Mucin	Mucin	Little mucin and extensive fibrosis	Mucin and lymphoceles	Mucin, fibrosis, and lymphocytes	Mucin	Mucin and fibrosis

All cases were considered locally advanced tumors on pre-CRT MR images (T3a, *n* = 2; T3c, *n* = 1; T3, *n* = 1; T4a, *n* = 3); most of them were with regional lymph node metastasis (*n* = 5) and MRF involvement (*n* = 5), while a few cases with EMVI involvement (*n* = 3). However, the post-CRT T-stage, EMVI, and MRF involvement of all the patients post-CRT were classified at the same level as the pre CRT on MR images.

### Comparison of MR Evaluation and Pathology TRG

MRI by a mandard standard only correctly evaluated for one good responder of pTRG 2 (accuracy, 14%), overestimated the tumor residue for all the patients of pCR, and underestimated the partial and poor responders. The accuracy of DWI was 71.4% (three good responders, one partial responder, and one poor responder), but DWI overestimated tumor residue for two good responders, shown in Table 2.

**Table 2:** Comparision of mrTRG (T2WI or T2WI + DWI) and pTRG.

**Patient**	**pTRG (score)**	**T2WI (score)**	**T2WI+DWI (score)**
Good-responsder1	1	5	1
Good-responsder2	2	2	1
Good-responsder3	1	4	2
Good-responsder4	1	3	5
Good-responsder5	1	3	1
Partial-responder	3	2	3
Poor-responder	5	4	5

## Discussion

The RSRCC, also called linitis plastica, is due to diffuse infiltrating of SRC and numerous desmoplastic reactions, especially in the gastric. However, two histology patterns of infiltration in rectal or colonal SRCC were described by WHO (1): (a) signet ring cells floating in copious pools of extracellular mucin; and (b) signet ring cells with a diffuse pattern of infiltration with minimal extracellular mucin, similar to diffuse-type, poorly cohesive carcinomas encountered in the stomach. In previous studies (14), circumferential thickening of the rectal mural with a concentric ring pattern (a target sign) with low SI on T2WI was considered a characteristic of rectal SRCC (corresponds to WHO Type b). In this study, classical targeted circumferential thickening of the rectal mural was noted in 71.4% (five of seven) patients. Slightly high SI with a thick-ring sign on DWI was also a characteristic sign of these SRCCs, and it corresponded to the diffuse distribution of SRCs in the entire mural. Multiple disconnected rings of low SI showed in the thickened mural either on T2WI or on enhanced phases were also the features, which correspond to separated mural layers and detached muscle bundles by infiltrated tumor tissue in histology, especially circular muscle. Besides, 28.1% (two of seven) patients mimicked mucinous adenocarcinoma without a target sign in our cohort, which was reported in only one case by the previous studies (17). Additionally, diffuse high SI and intermediate-high SI were more frequently showing rather than low SI on T2WI.

Hartman et al. (18) identified that mucin-poor signet ring cell carcinoma has a dismal prognosis with an aggressive clinical course, comparing to the mucin-rich SRCC in rectum. Similarly, in our study, the patient with low T2 SI, which represented mucin-poor tumor, showed worse CRT response than others. Three of four patients with high SI got a good response. It inferred that pre-treatment MRI might have the capacity to predict the CRT response and prognosis of SRCC, and it needs further study to confirm.

Mucinous degeneration was the dominant response to the CRT of SRCC, and it can be observed in all patients, especially the tumor with high SI on pre-CRT T2WI. It manifested as homogeneous high SI without enhancement, similarly as the form of MAC degeneration. However, the proportion of poorly differentiated adenocarcinoma components took place by extensive fibrosis, resulted in the moderated SI on pre-CRT T2WI, decreased markedly. At post-CRT DWI, most patients with good response showed thin-target signs due to the diffused tumor cell disappearance and cellular density decrease. Additionally, the mucin maintained made the mural remaining thickened, and even pCR was presented. Moreover, the ADC value of good responders increased more than partial/poor responders.

Tumor volume change is an important indicator of tumor treatment response. Our findings indicated that a significant increase in volume might indicate that the tumor is not responding well. The partial and poor responders showed more increase in tumor volume than the good responders, although tumor volume of the good responders did not show a marked reduction. We found that SRC was mostly mucus degeneration without markedly volume reduction. In contrast, the volume reduction of the adenocarcinoma component post-CRT was more apparent due to the tumor cell disappearance and fiber shrinkage. The study of Park et al. about the CRT of MAC had a similar result (11). Meanwhile, a large number of inflammatory cell infiltration in the submucosa of the lesion may attribute a slight increase of volume. However, we cannot evaluate the accuracy degeneration of SRCCs based on the volume change.

It seems that the TRG by mandard was also not satisfactory to evaluate the RSRCCs. It almost overestimated all tumors with good responses. Because either the stretched or broken muscle bundles or a large number of inflammatory reactions with lymphocyte proliferation and fibrosis were an equal and low signal on T2WI, which mimicked residue tumor floating in the mucin pools, so it was also challenging to evaluate CR. A previous study (11) of rectal mucinous adenocarcinoma mentioned that floating iso-signal tumor components could be distinguished in high-signal mucus lakes, and the regression of tumor components can be assessed separately. Its accuracy is about 40.7%, and this result was better than our result of SRCC. Because comparing to clustered with tumor cells of mucinous adenocarcinoma, SRCs are diffusely distributed, and it is more difficult to distinguish the tumor components from their mimickers mentioned above. However, the mimickers on T2WI did not show high SI on DWI, and it was easier to identify that the thick-ring sign turned into a thin-target sign on DWI in the good responders. Hence, it explains that T2WI with DWI has more accuracy than TRG by mandard in evaluating tumor response. However, a small number of scattered residual tumors have low cell density, which resulted in low/isointense on DWI, so one patient of pTRG Grade 2 tumors was underestimated as CR. Additionally, a good responder of CR on post-CRT pathology was estimated as a lot of residual tumor on both post-CRT T2WI and DWI. We thought the long interval (36 days) between post-CRT MRI and surgery might cause the mismatch of post-CRT MRI to pathology.

MRI staging post-CRT did not decrease in all patients on T2WI because of the inaccuracy of TRG.

### Limitations

There are several limitations in our study. Firstly, the cases enrolled were too few, and only one tumor showed classical image features as low SI on T2WI, while most tumors showed high SI. That may result in the bias of the better CRT response than previous pieces of research. Secondly, we did not use OS as the observation endpoint because two of seven patients were lost to follow-up.

## Conclusion

Circumferential thickening of the rectal mural with a target sign on T2WI and a thick-ring sign on DWI was the characteristic of rectal SRCC. Homogeneous high SI on T2WI and thin target sigh on DWI were the main MRI changes of RSRCC, which was corresponding to the mucinous regression and represented good response post-CRT. The mrTRG and tumor volume was not a reliable indicator to the pathology response. We considered that DWI should be added to T2WI to evaluate RSRCC response to CRT.

## Data Availability Statement

The original contributions presented in the study are included in the article/supplementary material, further inquiries can be directed to the corresponding author/s.

## Ethics Statement

The studies involving human participants were reviewed and approved by the Ethics Committee of the First Affiliated Hospital of Chongqing Medical University. The patients/participants provided their written informed consent to participate in this study.

## Author Contributions

YZ and QL: designed the study. YZ and YM: collected the data. QL and YM: analyzed the data. YZ, QL, and YM: prepared the manuscript. All authors read and approved the final manuscript.

## Conflict of Interest

The authors declare that the research was conducted in the absence of any commercial or financial relationships that could be construed as a potential conflict of interest.

## Publisher's Note

All claims expressed in this article are solely those of the authors and do not necessarily represent those of their affiliated organizations, or those of the publisher, the editors and the reviewers. Any product that may be evaluated in this article, or claim that may be made by its manufacturer, is not guaranteed or endorsed by the publisher.
